# Параметры электроэнцефалограммы у детей с врожденным гиперинсулинизмом, пролеченных по международному протоколу

**DOI:** 10.14341/probl13174

**Published:** 2023-02-25

**Authors:** Л. Р. Саракаева, Д. В. Рыжкова, Л. Б. Митрофанова, В. Г. Баиров, А. А. Сухоцкая, А. П. Смородин, Е. А. Ефтич, И. А. Кельмансон, И. Л. Никитина

**Affiliations:** Национальный медицинский исследовательский центр им. В.А. Алмазова; Национальный медицинский исследовательский центр им. В.А. Алмазова; Национальный медицинский исследовательский центр им. В.А. Алмазова; Национальный медицинский исследовательский центр им. В.А. Алмазова; Национальный медицинский исследовательский центр им. В.А. Алмазова; Национальный медицинский исследовательский центр им. В.А. Алмазова; Национальный медицинский исследовательский центр им. В.А. Алмазова; Национальный медицинский исследовательский центр им. В.А. Алмазова; Национальный медицинский исследовательский центр им. В.А. Алмазова

**Keywords:** врожденный гиперинсулинизм, электроэнцефалография, пароксизмальная активность, альфа-ритм, неонатальная гипогликемия

## Abstract

**ОБОСНОВАНИЕ:**

ОБОСНОВАНИЕ. Врожденный гиперинсулинизм (ВГИ) — редкое наследственное заболевание, основным проявлением которого являются персистирующие гипокетотические гипогликемии, способные привести к тяжелым необратимым неврологическим последствиям в отсутствие своевременной диагностики и адекватного лечения.

**ЦЕЛЬ:**

ЦЕЛЬ. Оценка нейрофизиологических характеристик центральной нервной системы детей, получивших лечение по поводу ВГИ по международному протоколу.

**МАТЕРИАЛЫ И МЕТОДЫ:**

МАТЕРИАЛЫ И МЕТОДЫ. В обсервационное ретроспективное проспективное исследование включены 73 пациента, получивших лечение по поводу ВГИ в соответствии с международным протоколом в клинике ФГБУ «НМИЦ им. В.А. ­Алмазова» в период с 2017 по 2022 гг. Медиана возраста пациентов составила 10 мес (6–58). Всем пациентам было выполнено комплексное обследование, включая проведение электроэнцефалографии.

**РЕЗУЛЬТАТЫ:**

РЕЗУЛЬТАТЫ. Из 73 пациентов с ВГИ, включенных в исследование, 35% (23) пациентов имели фокальную форму заболевания, 65% — нефокальную форму заболевания, из них диффузная форма отмечалась у 49% (39), атипичная — у 16% (11). Распределение по исходам заболевания: 100% пациентов с фокальной формой в исходе имели выздоровление. Среди пациентов с нефокальной формой медикаментозная компенсация отмечена у 60% пациентов с диффузной формой заболевания и у 36% пациентов с атипичной формой. Оперативное лечение получили 40% пациентов с диффузной формой и 64% пациента с атипичной формой.

При анализе данных электроэнцефалографии установлено, что пароксизмальная активность регистрировалась у 23 пациентов, что составило 32%, 50 (68%) пациентов пароксизмальной активности не имели. Диффузные изменения отмечены у 47 (64%) пациентов, 26 (36%) пациентов их не имели. Путем построения кривых Каплана–Мейера установлено, что альфа-ритм формируется достоверно (р=0,026) раньше у пациентов с фокальной формой.

**ЗАКЛЮЧЕНИЕ:**

ЗАКЛЮЧЕНИЕ. Грубые нарушения нейрофизиологических параметров у пациентов с ВГИ, получивших лечение по современному международному протоколу, встречаются относительно редко. Удалось установить ассоциацию наиболее раннего формирования альфа-ритма с фокальной формой ВГИ.

## Abstract (en)

**BACKGROUND:**

BACKGROUND: Congenital hyperinsulinism (CHI) is a rare life-threatening disease characterised by persistent hypoglycaemia as a result of inappropriate insulin secretion, which can lead to irreversible neurological defects in infants.

**AIM:**

AIM: To evaluate neurophysiological characteristics of central nervous system in children with congenital hyperinsulinism treated according to the international protocol in Russian Federation.

**MATERIALS AND METHODS:**

MATERIALS AND METHODS: Our retrospective, prospective cohort study included 73 patients who received treatment for CHI according to the current international protocol at different departments of the Almazov National Medical Research Centre from 2017 to 2022. All patients underwent a comprehensive examination, including electroencephalography (EEG).

**RESULTS:**

RESULTS: Among 73 patients with CHI, 35% (23) had focal form of the disease, 65% had non-focal form (49% (39) — diffuse form, 16% (11) — atypical form). All patients with focal form of CHI had a recovery as an outcome.

Analysing the EEG data we found that paroxysmal activity was recorded in 23 patients (32%), 50 patients did not have paroxysmal activity (68%). Diffuse changes were observed in 47 patients (64%), whereas 26 patients (36%) were absent of it. By constructing Kaplan-Meier curves we found that the alpha rhythm is formed significantly (p=0.026) earlier in patients with a focal form of CHI.

**CONCLUSION:**

CONCLUSION: CHI patients treated according to the international guidelines in Russian Federation show rather positive neurological outcome. We established that alpha rhythm earliest formation is associated with focal form of CHI.

## ОБОСНОВАНИЕ

Врожденный гиперинсулинизм (ВГИ) — тяжелое наследственное заболевание, основным проявлением которого являются персистирующие гипогликемические состояния, представляющие серьезную угрозу состоянию центральной нервной системы (ЦНС) детей раннего возраста [1]. ВГИ относится к категории редких заболеваний, несмотря на то что выявляемость ВГИ в Российской Федерации существенно увеличилась в последние годы и составляет, по данным М.А. Меликян, 1:50 638 живых новорожденных, что сопоставимо с данными мировых регистров [2].

Главной отличительной чертой гипогликемии при ВГИ является подавление в условиях гиперинсулинемии липолиза и кетогенеза — синтеза альтернативных источников энергии для нервной системы. Это явление объясняется тем, что инсулин обладает анаболическим свойством в отношении жировой ткани, препятствуя ее расщеплению и превращению в кетоновые тела в условиях его повышенной концентрации. Лишенная, таким образом, и основного, и альтернативного энергетического субстрата ЦНС оказывается в крайне уязвимом положении, что является причиной тяжелых неврологических исходов у данной категории пациентов [3–5].

Благодаря существенному расширению представлений о ВГИ с точки зрения генетики, патоморфологии, патофизиологии и совершенствованию международного протокола по ведению пациентов с ВГИ в последние два десятилетия удалось значимо преобразить потенциальные исходы ВГИ и сократить долю пациентов, имеющих выраженные неврологические последствия [6–8].

В отношении неврологических исходов детей с ВГИ продолжают проводиться исследования, направленные на оценку параметров психомоторного развития (ПМР); продолжает изучаться влияние различных факторов на неврологические исходы с определением наиболее значимых в отношении неврологического прогноза. Помимо оценки показателей ПМР, для более объективного изучения состояния ЦНС и соответствия ее характеристик возрастным нормативам интересным представляется определение нейрофизиологических параметров ЦНС, что в полной мере позволяет обеспечить электроэнцефалография (ЭЭГ) — неинвазивный метод исследования функционального состояния головного мозга путем регистрации его биоэлектрической активности. В частности, ЭЭГ широко применяется в неонатологии для определения функциональной зрелости ЦНС у недоношенных детей [9][10].

Картина биоэлектрической активности головного мозга отражает морфофункциональное состояние ЦНС ребенка, в каждый из возрастных периодов она имеет свои особенности, определенные сроки формирования различных паттернов ЭЭГ и ритмической активности, что позволяет определить соответствие нейрофизиологических характеристик ЦНС установленным возрастным параметрам.

К 3 мес жизни наблюдаются исчезновение неонатальных паттернов ЭЭГ и появление заднего основного (доминантного) ритма — предшественника альфа-ритма, что свидетельствует о предпосылках формирования более зрелых паттернов, наблюдаемых у взрослых. Альфа-ритм демонстрирует реакцию на открытие и закрытие глаз: блокируется при открытии глаз и активируется при закрытии глаз, поэтому крайне важно соблюдать технику проведения процедуры ЭЭГ с применением окклюзии глаз для корректного определения доминирующей ритмической активности. Задний основной (доминантный) альфа-ритм становится более стабильным в возрасте 5 мес, со временем увеличивается его частота, и соответственно частоте стабилизируется амплитуда [11–13].

Поражения ЦНС, в том числе метаболические вследствие гипогликемии, могут нарушать функцию ЦНС и вызывать регрессию развития, которая может выражаться в возвращении к менее зрелому паттерну ЭЭГ. Наряду с изменениями в картине биоэлектрической активности в условиях метаболического неблагополучия на ЭЭГ может формироваться пароксизмальная активность. Таким образом, представило интерес проведение углубленного анализа ЭЭГ у пациентов с ВГИ с точки зрения оценки функционального состояния ЦНС и прогноза неврологических исходов у данной категории пациентов. На данном этапе исследования изучались следующие нейрофизиологические параметры: наличие пароксизмальной активности, диффузных изменений и сроки формирования альфа-ритма.

## ЦЕЛЬ ИССЛЕДОВАНИЯ

Целью нашего исследования стали изучение состояния нервной системы пациентов, получивших лечение по поводу ВГИ по международному протоколу, с точки зрения нейрофизиологических характеристик, а также анализ влияния различных факторов, связанных с заболеванием, на нейрофизиологический статус пациента.

## МАТЕРИАЛЫ И МЕТОДЫ

Место и время проведения исследования

Исследование проведено на базе Федерального государственного бюджетного учреждения «Национальный медицинский исследовательский центр им. В.А. Алмазова» Минздрава России. В исследование включены 73 пациента с ВГИ, получившие стационарное лечение в соответствии с международным протоколом. Набор пациентов проводился в период с 2017 по 2021 гг.

Критерии соответствия

Критерии включения: возраст от 0 до 18 лет, подтвержденный диагноз ВГИ, стационарное лечение в ФГБУ «НМИЦ им. В.А. Алмазова» с 2017 г. по международному протоколу, наличие согласия ребенка и/или законного представителя на участие в исследовании.

Критерии исключения: гипогликемии негиперинсулинемического генеза, наличие другой уточненной патологии ЦНС, отказ от участия в исследовании.

Дизайн исследования

Исследование выполнено в дизайне одноцентрового обсервационного проспективного.

Методы

Все пациенты, включенные в исследование, получили стационарное лечение по поводу ВГИ в рамках рутинной клинической практики в соответствии с актуальным международным протоколом, включающим оценку общесоматического состояния пациента, лабораторное обследование с определением ряда биохимических показателей для лабораторной верификации диагноза путем определения уровня глюкозы, инсулина и С-пептида в одной пробе плазмы крови, радионуклидное обследование с применением ПЭТ-КТ с [18F]-фторДОФА (позитронно-эмиссионной компьютерной томографии с 18F-L-диоксифенилаланином) [14]. Пациенты со значениями панкреатического индекса по ПЭТ-КТ с [18F]-фторДОФА выше 1,5, что с высокой вероятностью свидетельствует о фокальной форме заболевания, направлялись на хирургическое лечение ВГИ; также хирургическое лечение получали дети с фармакорезистентными нефокальными формами. В ходе гистологического исследования устанавливался окончательный морфологический вариант заболевания. Комплексное изучение неврологического статуса включало оценку ПМР и нейрофизиологических характеристик пациентов с ВГИ методом ЭЭГ через 6 мес и более после установления диагноза и начала специфического лечения. ЭЭГ выполнялось на энцефалографе NICOLET ONE (США) в базовой стационарной комплектации. Использовалась международная система наложения электродов «10–20», 19-канальная электродная система в виде шапочки. Сопротивление электродов менее 5 кОм. Полоса пропускания 0,5–70 Гц. Процедура ЭЭГ — регистрация, обработка и анализ ЭЭГ — осуществлялась по общепринятой методике в соответствии со стандартизированным протоколом [15].

В качестве оцениваемых параметров исследовались доминирующая ритмическая активность, возраст возникновения альфа-ритма или альфа-подобной активности, наличие пароксизмальной активности и диффузных изменений. Затем было оценено влияние различных факторов, ассоциированных с заболеванием, на нейрофизиологические характеристики пациентов, получивших лечение по поводу ВГИ. С точки зрения возможной ассоциации с исходом заболевания в данном исследовании были изучены следующие факторы: форма заболевания (фокальная или иная), возраст дебюта заболевания и верификации диагноза, минимальный уровень гликемии, максимальная углеводная нагрузка.

Статистический анализ

Статистическая обработка данных выполнена с использованием программного обеспечения Jamovi 2.2.4. Первоначальная обработка данных произведена с помощью стандартной описательной статистики. Аналитическая обработка данных проводилась методом многофакторного регрессионного и корреляционного анализа. Различия были признаны статистически значимыми при вероятности ошибки менее 5% (p<0,05). Для оценки влияния отдельных клинических характеристик на сроки начала формирования альфа-подобной активности на ЭЭГ детей использован анализ типа «продолжительности жизни» (метод Каплана–Мейера). Срок появления альфа-активности на ЭЭГ в процессе динамического наблюдения за ребенком рассматривался в качестве зависимой переменной. Изученные клинические характеристики, в том числе форма заболевания, рассматривались в качестве объясняющих переменных. В тех случаях, когда на протяжении всего периода динамического наблюдения за ребенком начало формирования альфа-активности не было зафиксировано, наблюдение расценивалось как цензурированное [16].

Этическая экспертиза исследования проведена Этическим комитетом ФГБУ «НМИЦ им. В.А. Алмазова» Минздрава РФ 13 декабря 2021 г. Протокол заседания Этического комитета № 12-21-01С. Родители всех обследованных пациентов представили информированное согласие на участие в данном исследовании.

## РЕЗУЛЬТАТЫ

В исследование включены 73 пациента с верифицированным диагнозом ВГИ. Медиана возраста детей, включенных в исследование, составила 10 (6–58) мес. Среди включенных в исследование пациентов отмечалось следующее распределение по полу: 43 (59%) девочки, 30 (41%) мальчиков (рис. 1). По результатам, полученным при проведении ПЭТ-КТ с [18F]-фторДОФА, фокальная форма диагностирована у 25 (35%) пациентов, нефокальная форма — у 48 (65%) (рис. 2). Некоторые результаты ПЭТ-КТ с [18F]-фторДОФА представлены на рисунке 3. У оперированных пациентов форма ВГИ была подтверждена при гистологическом исследовании (рис. 4). Все пациенты с фокальной формой получили хирургическое лечение, в исходе которого у 100% имело место выздоровление. Пациенты с нефокальной формой ВГИ при наличии чувствительности к инсулиностатическим препаратам получали консервативную терапию. В случае фармакорезистентной нефокальной формы пациенты получали оперативное лечение, объем операции определялся по результатам интраоперационной биопсии. В исходе заболевания у пациентов с нефокальной формой 30 пациентов имели медикаментозную компенсацию, 3 пациента в исходе оперативного лечения нефокальной формы — выздоровление, у 8 пациентов развился диабет и у 7 пациентов отмечен рецидив заболевания, потребовавший возобновления инсулиностатической терапии в послеоперационном периоде. Распределение пациентов по исходу заболевания представлено на рисунке 5.

В соответствии с дизайном исследования в качестве оцениваемых параметров ЭЭГ исследовались доминирующая ритмическая активность, возраст возникновения альфа-ритма или альфа-подобной активности, наличие пароксизмальной активности и диффузных изменений.

По результатам ЭЭГ через 6 мес и более после окончания лечения по поводу ВГИ у 40 (55%) пациентов регистрировался тета-ритм, у 16 (22%) — альфа-ритм, у 9 (12%) — полиморфная активность и у 8 (11%) — дельта-ритм (рис. 6). Пароксизмальная активность регистрировалась у 23 (32%), 50 (68%) пациентов пароксизмальной активности не имели (рис. 7); 47 (64%) пациентов имели диффузные изменения, 26 (36%) — их не имели (рис. 8). Следующим этапом исследования стало проведение анализа влияния основных факторов, связанных с заболеванием, на нейрофизиологические характеристики пациентов с ВГИ. Методом построения кривой Каплана–Мейера, где в качестве функции выживаемости было задано появление альфа-ритма, установлено, что у пациентов с фокальной формой возраст возникновения альфа-ритма был более ранним, а следовательно, более физиологичным по сравнению с пациентами с нефокальными формами ВГИ. Таким образом, выявлена прогностическая значимость фокальной формы ВГИ в отношении лучших, а именно более близких к физиологическим, сроков формирования альфа-ритма (р=0,026) (рис. 9).

Что касается формирования пароксизмальной активности, не выявлено достоверного влияния факторов, связанных с заболеванием, на ее регистрацию: формы заболевания (р=0,579), возраста дебюта (р=0,452) и верификации заболевания (р=0,562), минимального уровня гликемии (р=0,740), максимальной углеводной нагрузки (р=0,788).

Подобные результаты получены при анализе влияния факторов, связанных с заболеванием, на формирование диффузных изменений ЭЭГ: формы заболевания (р=0,594), возраста дебюта (р=0,632) и верификации заболевания (р=0,659), минимального уровня гликемии (р=0,951), максимальной углеводной нагрузки, необходимой для поддержания целевого уровня гликемии (р=0,066).

**Figure:**
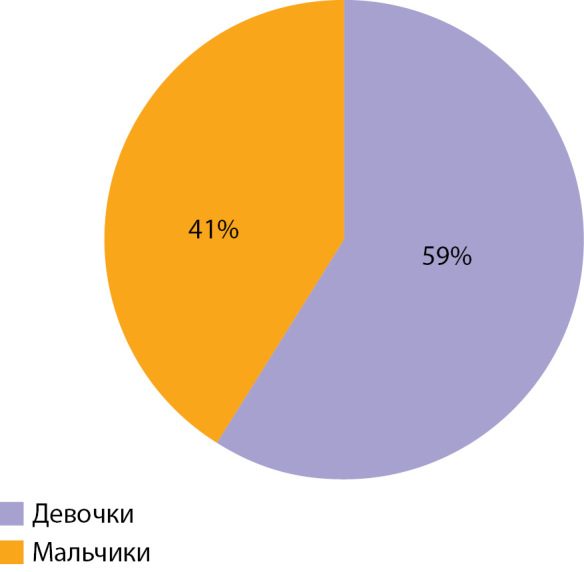
Рисунок 1. Распределение пациентов, включенных в исследование, по полу.

**Figure:**
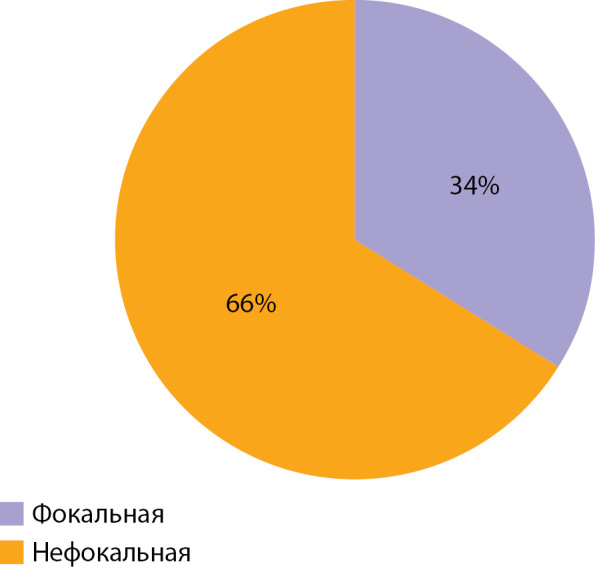
Рисунок 2. Распределение пациентов, включенных в исследование, по форме заболевания.

**Figure:**
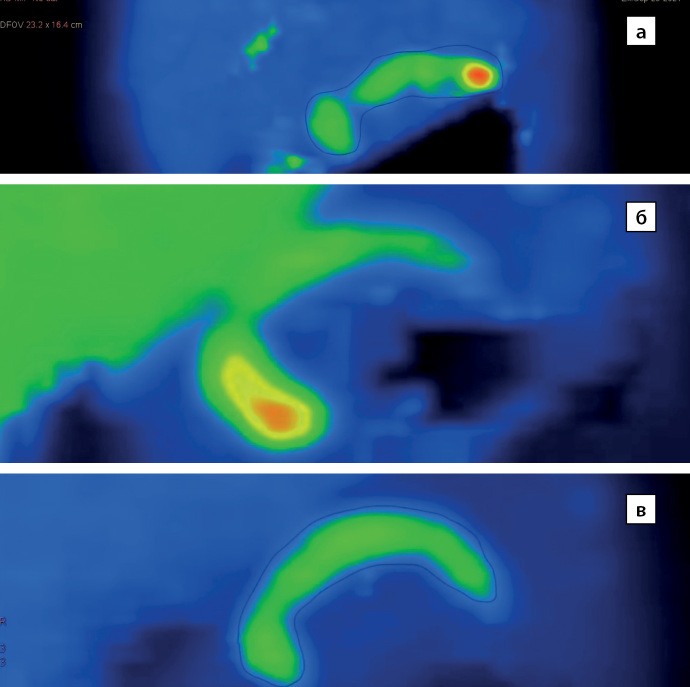
Рисунок 3. Результаты ПЭТ-КТ с [18F]-фторДОФА: а — фокальная форма ВГИ с локализацией очага накопления радиофармпрепарата в хвосте поджелудочной железы; б — фокальная форма ВГИ с локализацией очага накопления радиофармпрепарата в головке поджелудочной железы; в — диффузная форма ВГИ — захват радиофармпрепарата равномерен (архив Рыжковой Д.В.).

**Figure:**
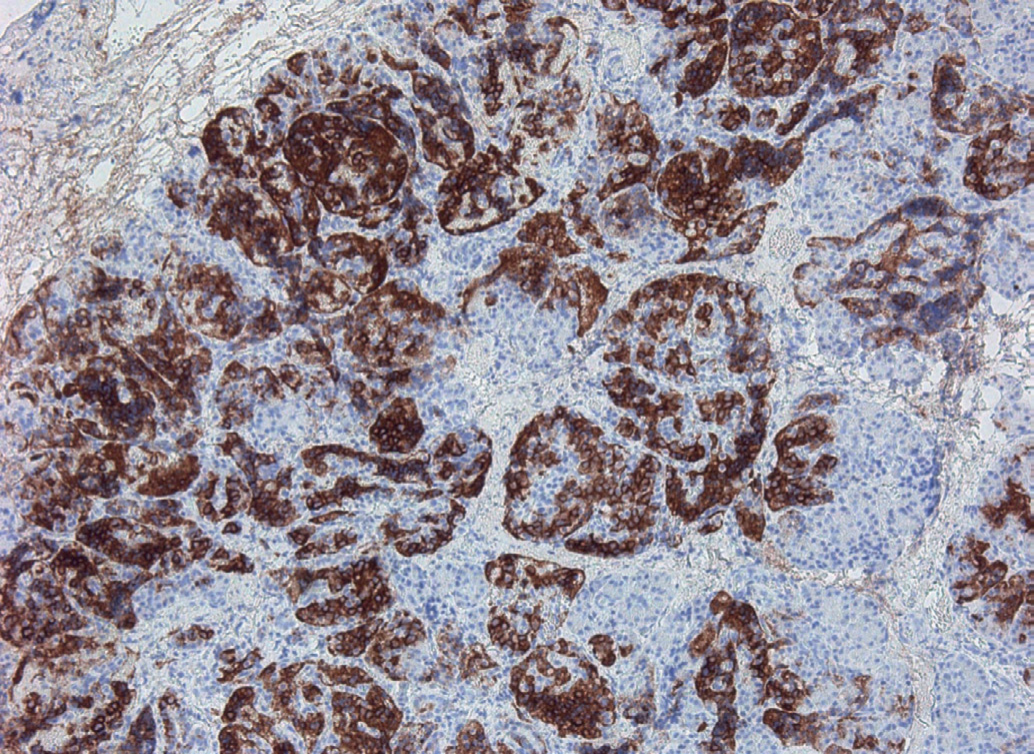
Рисунок 4. Результаты гистологического исследования — фокальная форма ВГИ (архив Митрофановой Л.Б.).

**Figure:**
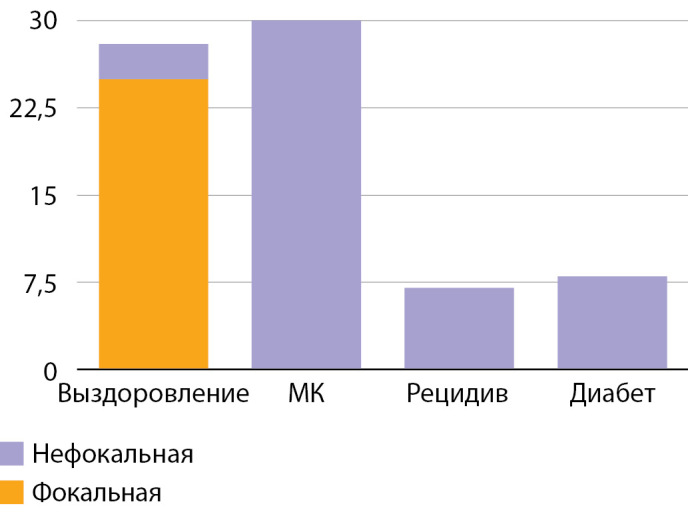
Рисунок 5. Распределение пациентов по метаболическому исходу заболевания. МК — медикаментозная компенсация.

**Figure:**
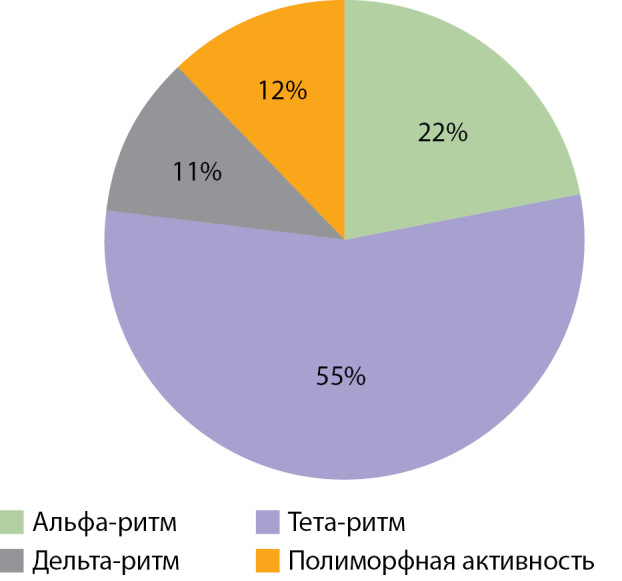
Рисунок 6. Распределение пациентов по доминирующей ритмической активности.

**Figure:**
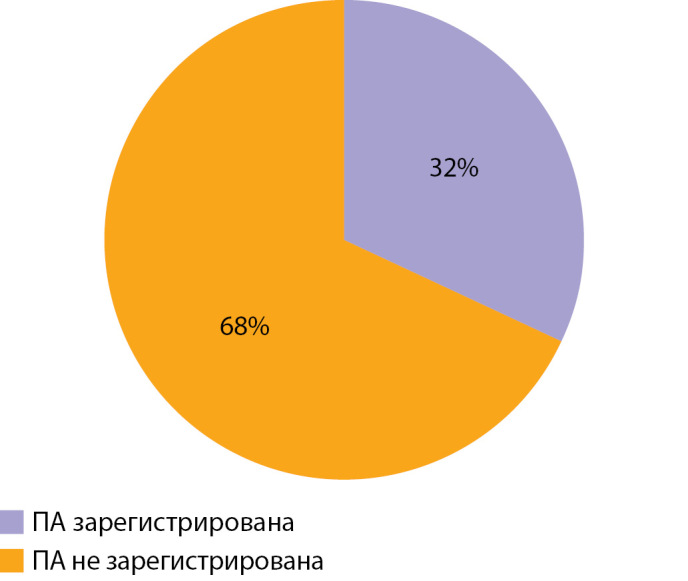
Рисунок 7. Распределение пациентов по наличию пароксизмальной активности (ПА) на ЭЭГ.

**Figure:**
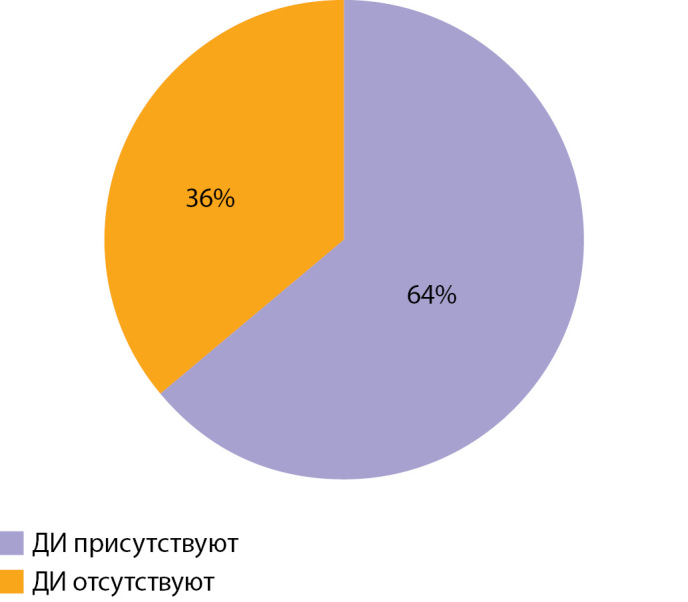
Рисунок 8. Распределение пациентов по наличию диффузных (ДИ) изменений на ЭЭГ.

**Figure:**
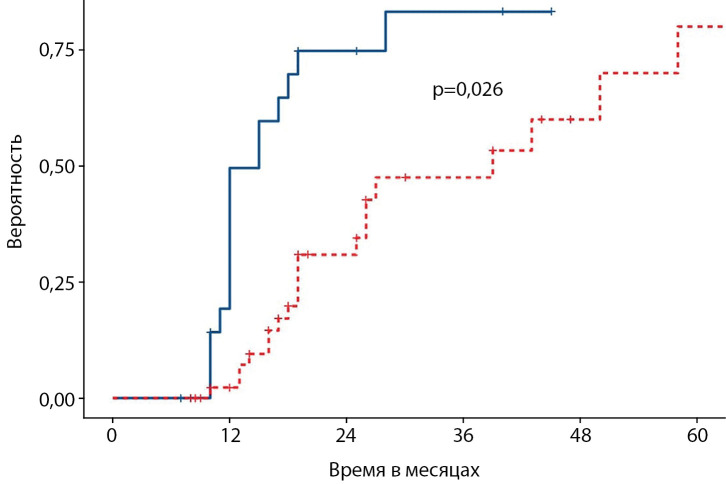
Рисунок 9. Прогнозирование сроков формирования альфа-ритма по ЭЭГ в зависимости от формы ВГИ (метод Каплана–Майера).

## ОБСУЖДЕНИЕ

Репрезентативность выборок

Поскольку ВГИ относится к редким заболеваниям, формирование многочисленной выборки представляет собой трудную задачу, тем не менее в нашем исследовании приняли участие 73 ребенка с данной патологией, прошедших обследование в специализированном центре, что позволило обеспечить достаточную численность выборки, сопоставимой с числом пациентов, задействованных в аналогичных исследованиях. Предварительный расчет размера выборки не проводился, однако при ретроспективной оценке количества включенных в исследование пациентов с помощью программного обеспечения G*power установлено, что размер выборки обеспечивает достоверность полученных результатов.

Сопоставление с другими публикациями

Благодаря значимым успехам в совершенствовании протокола диагностики и лечения детей с ВГИ результаты в отношении неврологических исходов, представленные в мировой литературе, с течением времени приобретают все более оптимистичный характер. До внедрения в клиническую практику ПЭТ-КТ с [18F]-фторДОФА, позволившей осуществлять неинвазивное определение формы заболевания, следовательно, обеспечивать таргетное хирургическое лечение в случае фокальной формы заболевания, доля пациентов с неблагоприятными неврологическими исходами являлась превалирующей, при этом относительно более благоприятный неврологический исход отмечался у пациентов, обладающих чувствительностью к инсулиностатической терапии [17–20].

С внедрением современного протокола оказания помощи пациентам с ВГИ значимо сократилось число неблагоприятных неврологических исходов, в то же время в ряде исследований была обнаружена ассоциация развития неблагоприятных неврологических последствий с более поздним поступлением пациента в центр экспертного уровня, а значит, удлинение процесса постановки диагноза и назначения адекватного лечения [21–23]. В нашем исследовании также проводились изучение риска формирования пароксизмальной активности и диффузных изменений, определение сроков становления альфа-ритма у детей в зависимости от клинических особенностей заболевания. Было установлено, что риск формирования патологических изменений на ЭЭГ не зависел от ряда обозначенных в нашем исследовании клинических факторов. Вероятно, данное наблюдение можно объяснить тем, что все пациенты, включенные в наше исследование и получившие лечение в ФГБУ «НМИЦ им. В.А. Алмазова», имели сравнительно небольшие и сопоставимые сроки маршрутизации, таким образом, при прочих относительно равных условиях наиболее значимое модифицирующее влияние на неврологический исход имела форма заболевания. Отдельным аспектом нашего исследования является оценка влияния факторов, ассоциированных с заболеванием, на сроки формирования альфа-ритма: в доступной литературе подобных данных обнаружить не удалось.

Полученные нами данные укладываются в основную парадигму представлений о ВГИ, существующую в мировом научном сообществе: с течением времени неврологические исходы детей с данной патологией значимо улучшаются, что связано с совершенствованием диагностических и лечебных возможностей, улучшением информированности об этом заболевании, обуславливающим более раннее выявление детей с ВГИ и ранний старт специфической терапии.

Принципиально новым открытием стало установление того факта, что при условиях, когда подавляющее большинство детей с ВГИ имеют раннюю верификацию диагноза и своевременный старт терапии, ключевую роль в отношении неврологического исхода и нейрофизиологических параметров пациентов имеет форма заболевания: дети с фокальной формой ВГИ имели более ранние, а следовательно, более физиологичные сроки формирования альфа-ритма. Что касается состояния ЦНС у детей с нефокальными формами ВГИ, в литературе обсуждается не связанный с гипогликемическими состояниями генез поражения ЦНС, а именно влияние определенных патогенных вариантов в генах, экспрессированных не только в поджелудочной железе, но и в структурах ЦНС, определяющих нарушение дифференцировки и формирования ее компонентов [24][25].

Направления дальнейших исследований

С учетом постоянно растущего количества пациентов с ВГИ, наблюдающихся в «НМИЦ им. В.А. Алмазова», планируются дальнейшее расширение размера когорты пациентов, включенных в исследование, а также динамическое наблюдение за показателями ПМР и нейрофизиологическими параметрами растущих пациентов с ВГИ с оценкой долгосрочных результатов лечения по новому (международному) протоколу в России.

## ЗАКЛЮЧЕНИЕ

В ходе нашего исследования установлено, что у пациентов с фокальной формой ВГИ сроки появления альфа-ритма были достоверно более ранними в сравнении с пациентами с нефокальными формами ВГИ. Принимая во внимание тот факт, что раннее формирование альфа-ритма является более физиологичным, сделано заключение о том, что фокальная форма заболевания является прогностической в отношении более благоприятного неврологического исхода в целом.

Полученные результаты во многом согласуются с данными зарубежных исследований; не исключено, что сравнительно худшие показатели нейрофизиологических характеристик у пациентов с нефокальными формами ВГИ обусловлены не столько метаболическими последствиями гипогликемии, сколько самостоятельным влиянием определенных генетических вариантов, ассоциированных с нарушением дифференцировки и формирования ЦНС вследствие экспрессии генов, вовлеченных в патологический процесс, не только в поджелудочной железе, но и в структурах ЦНС.

Что касается других нейрофизиологических параметров ЦНС — пароксизмальной активности и диффузных изменений, не установлено достоверного влияния таких факторов, ассоциированных с заболеванием, как форма заболевания, возраст дебюта и верификации заболевания, минимальный уровень гликемии, максимальная углеводная нагрузка, на их представленность в биоэлектрической картине детей с ВГИ.

## ДОПОЛНИТЕЛЬНАЯ ИНФОРМАЦИЯ

Источники финансирования. Работа выполнена по инициативе авторов без привлечения финансирования.

Конфликт интересов. Авторы декларируют отсутствие явных и потенциальных конфликтов интересов, связанных с содержанием настоящей статьи.

Участие авторов. Саракаева Л.Р. — существенный вклад в концепцию и дизайн исследования, написание статьи; Рыжкова Д.В. — существенный вклад в концепцию и дизайн исследования, написание и внесение в рукопись существенной правки с целью повышения научной ценности статьи; Митрофанова Л.Б. — существенный вклад в анализ данных и интерпретацию результатов, внесение в рукопись существенной правки с целью повышения научной ценности статьи; Баиров В.Г. — существенный вклад в получение и анализ данных, участие в написании статьи; Сухоцкая А.А. — существенный вклад в получение и анализ данных, участие в написании статьи; Смородин А.П. — существенный вклад в получение и анализ данных; Ефтич Е.А. — существенный вклад в получение и анализ данных, участие в написании статьи; Кельмансон И.А. — существенный вклад в анализ данных и интерпретацию результатов, внесение в рукопись существенной правки с целью повышения научной ценности статьи; Никитина И.Л. — существенный вклад в концепцию и дизайн исследования, написание и внесение в рукопись существенной правки с целью повышения научной ценности статьи.

Все авторы одобрили финальную версию статьи перед публикацией, выразили согласие нести ответственность за все аспекты работы, подразумевающую надлежащее изучение и решение вопросов, связанных с точностью или добросовестностью любой части работы.
